# Preparation and Characterization of Hydroxyapatite Coating on AZ31 Magnesium Alloy Induced by Carboxymethyl Cellulose-Dopamine

**DOI:** 10.3390/ma14081849

**Published:** 2021-04-08

**Authors:** Yanxia Yang, Yuanzhi Wu, Yu Wei, Tian Zeng, Baocheng Cao, Jun Liang

**Affiliations:** 1 School of Stomatology, Lanzhou University, Lanzhou 730000, China; yangyx18@lzu.edu.cn (Y.Y.); wuyzh14@lzu.edu.cn (Y.W.); weiyu19@lzu.edu.cn (Y.W.); zengt19@lzu.edu.cn (T.Z.); 2 State Key Laboratory of Solid Lubrication, Lanzhou Institute of Chemical Physic, Chinese Academy of Sciences, Lanzhou 730000, China

**Keywords:** carboxymethyl cellulose-dopamine, magnesium alloys, biomimetic mineralization, hydroxyapatite, corrosion resistance, cytotoxicity

## Abstract

Magnesium and its alloys have become potential implant materials in the future because of light weight, mechanical properties similar to natural bone, good biocompatibility, and degradability in physiological environment. However, due to the rapid corrosion and degradation of magnesium alloys in vivo, especially in the environment containing chloride ions, the application of magnesium alloys as implant materials has been limited. Therefore, improving the corrosion resistance of magnesium alloy and ensuring good biocompatibility is the main focus of the current research. In this study, hydroxyapatite coating was prepared on magnesium alloy surface using carboxymethyl cellulose-dopamine hydrogel as inducer to improve corrosion resistance and biocompatibility. Surface characterization techniques (scanning electron microscopy, Fourier-transformed infrared spectroscopy, energy dispersive X-ray spectroscopy- and X-ray diffraction) confirmed the formation of hydroxyapatite on the surface of AZ31 alloy. Corrosion resistance tests have proved the protective effect of Carboxymethyl cellulose-Dopamine/hydroxyapatite (CMC-DA/HA) coating on the surface of AZ31 alloy. According to MC3T3-E1 cell viability and Live/Dead staining, the coating also showed good biocompatibility. The results will provide new ideas for the biological application of magnesium alloys.

## 1. Introduction

As temporary orthopedic implant materials, magnesium and its alloys have great advantages because of comparable mechanical properties and good biocompatibility with natural bone [1,2,3,4]. In addition, compared with other traditional implant materials, the degradability of magnesium alloy avoids the secondary removal of implants [5]. However, magnesium’s inherent low corrosion resistance has become one of the main obstacles, which may cause hydrogen precipitation and alkalization of the solution in the physiological environment [6,7,8], and the rapid degradation of magnesium alloy can cause local stress concentration and reduction in the mechanical strength, thereby shortening the life of the implant. Therefore, many studies are devoted to improving the corrosion resistance of magnesium alloys through surface treatment [8,9,10].

Calcium phosphate (CaP) coating is often used as the modification of biological implant materials because of its excellent properties [6,11]. As the major inorganic composition in natural bone, it has good biocompatibility, bone conductibility, and biological activity [12]. As a kind of the calcium phosphate, hydroxyapatite (HA, Ca_10_(PO_4_)_6_(OH)_2_) has a pH of 4–12 at room temperature [13]. It is chemically stable under physiological conditions [14]. HA has obvious advantages in formative composites. Due to its conductive property, HA can be tightly combined with bone in a short time, which not only plays a major role in improving the mechanical properties of composites, but also provides a good environment for the proliferation and adhesion of osteoblasts [15,16]. At present, there are many methods to form HA on the surfaces of magnesium alloys, including hydrothermal methods [17,18], sol–gel methods [19,20], and biomimetic mineralization methods [21]. The biomimetic mineralization method can simulate the mineralization process of physiological apatite in a natural environment [22,23]. It has the characteristics of environmental friendliness and a simple process. At the same time, it has the characteristics of osteogenesis and the ability to combine with bone growth-stimulating factor [24].

According to the different pretreatment, there are many methods of biomimetic mineralization. Among them, self-assembly molecular layer method is a newly developed and effective method. It can synthesize HA directly on the surface of biopolymers in physiological environment to produce HA/biopolymer composites with excellent properties. Some studies have shown that the combination of HA and biopolymer greatly increased the mechanical properties and improved the protein adsorption capacity [25,26]. In addition, biopolymer with polar functional groups (such as −COOH, −OH) are very popular because these functional groups have a greater affinity for Ca^2+^ and lead to the formation of HA in solution [27,28].

Carboxymethyl cellulose (CMC) is extensively applied in pharmaceutical, food, and other industries [29]. It has a high solubility and a low cost, and it is also nontoxic and biodegradable [30]. More importantly, there are many carboxyl and hydroxyl groups in CMC, which can be cross-linked and chemically combined under certain conditions, playing a driving role in the mineralization process and self-assembly of natural bone [31]. Some studies [32,33] have confirmed that HA nanocomposites can be prepared using CMC to simulate the natural nucleation and growth process. However, the adhesion strength of CMC is low, and it is urgent to improve the adhesion performance through modification [34].

Dopamine (DA) is a kind of amino acid, which can interact strongly with other materials through chemical and physical interaction and shows high adhesion on various materials [35]. Because of its strong adhesion to the material surface, DA is considered to be an important component of the mussel adhesion protein [36]. Furthermore, it has high cell affinity and can be used in biomedical applications [14], and the dopamine-modified materials have been widely used in tissue engineering [37,38,39]. In addition, the hydroxyl groups in PDA formed by self-polymerization of DA monomers can be used as a second-order reaction platform to further modify the active molecules on the surface of the metal substrate [40]. With this background, we sought to introduce DA into CMC to enhance the bonding properties to magnesium alloys and ensure good biocompatibility.

In this study, a biomimetic method involved of CMC-DA hydrogels to prepare uniform and bioactive CMC-DA/HA coating on Mg alloys was first present. The carboxyl and hydroxyl groups in the CMC-DA hydrogels can act as nucleation centers to induce calcium and phosphorus deposition. At the same time, this composite hydrogel is also a degradable material with high solubility, and theoretically has good biocompatibility. We will systematically evaluate the corrosion resistance and cytotoxicity of modified magnesium alloys in order to provide a basis for the clinical application of modified magnesium alloys.

## 2. Materials and Methods

### 2.1. Preparation of AZ31 Samples

AZ31 Mg alloy (Al 2.5–3.0, Zn 0.7–1.3, Mn > 0.20 and the balance Mg) was purchased from Kuang Yu Metal co., Ltd., Dongguan, China. The final dimension of the samples is 1 mm × 10 mm × 10 mm. They were polished with SiC sand papers and washed by ultrasonic in 100% ethanol and deionized water for 0.5 h. Before experiment, all AZ31 samples were immersed into 5 M NaOH for 24 h to passivate the surfaces.

### 2.2. Synthesis of Carboxymethyl Cellulose–Dopamine (CMC-DA) Hydrogels

First, 0.25 g CMC was put into the phosphate buffer solution (PB solution, pH = 5, NaCl = 1.56 g/L, NaH_2_PO_4_ = 0.584 g/L) of 40 mL and stirred in a water bath of 30 °C for 24 h. When CMC was completely dissolved in PB solution, adjusted pH to about 5.0. Before the reaction, N_2_ was continuously injected into the solution for 30 min to remove air from the solvent.

N-Hydroxy succinimide (NHS), 1-(3-Dimethylaminopropyl)-3-ethylcarbodiimide hydrochloride (EDC), and DA were weighed with specified proportions. First, NHS and DA were added sequentially to the CMC solution. At the same time, EDC was dissolved in 10 mL of PB buffer solution, after which it was added to mixture of NHS and DA at a speed of 2.5 mL/2 h using a constant-pressure funnel. The reaction was carried out at 30 °C for 24 h under nitrogen and in a dark environment.

After the reaction, the solution was poured into anhydrous ethanol to separate the crude product. And then the crude product was fully dissolved in the PB buffer solution and placed in a dialysis bag with a molecular weight of 8000–14,000 Da. It was dialyzed first in the PB buffer solution with pH = 5 for 3 days and then in deionized water for an additional 3 days. Finally, the sample was dried by a freeze-drier (FD-1A-50, Shanghai Xinnuo Instrument Group Co., Ltd. Shanghai, China).

### 2.3. Preparation of CMC-DA Films on AZ31 Surfaces

Before the experiment, 0.15 g of the CMC-DA sample was dissolved in 25 mL of PB solution (pH = 5). Then, the prepared CMC-DA hydrogel was dropped onto the surface of the AZ31 substrate and the sample was rotated by a spin coating machine (Model kw-4 desktop, Xin Youyan Electronic Technology Co., Ltd., Beijing, China).

### 2.4. CMC-DA-Assisted Hydroxyapatite (HA) Formation

A calcium–phosphorus (CaP) solution was prepared as described by Cui et al. [41]. The final concentration of the CaP solution was as follows: 14 mM Ca(NO_3_)_2_, 8.4 mM NaH_2_PO_4_, and 4 mM NaHCO_3_. The CMC-DA-coated AZ31 was immersed into the CaP solution and incubated at 37 °C for 48 h to induce biomimetic mineralization of hydroxyapatite. In order to better observe the experimental results, the uncoated magnesium alloy was also put into the solution for control.

### 2.5. Surface Characterization

The freeze-dried CMC-DA was dissolved in PB solution with pH = 5 to prepare 0.25 mg/mL solution. The full spectrum was scanned by ultraviolet–visible (UV–VIS; UV-2800A, UNIC, Shanghai, China) and the absorbance was determined at 280 nm; CMC-DA and KBr were mixed and pressed into tablets. The spectrum of 4000–400 cm^−1^ was collected at room temperature and humidity of about 65% using Fourier-transform infrared (FTIR; NEXUS 670, Nicolet, WI, USA).

The surface morphologies and the cross-sectional morphologies of the coatings were viewed by scanning electron microscopy (SEM, nSM–5600LV, JEOL, Tokyo, Japan). However, the constituent elements of the surface were analyzed by energy-dispersive X-ray spectroscopy (EDS, Hitachi S-4800, Hitachi, Ltd., Tokyo, Japan) operating at 20 kV. Detecting the newly synthesized chemical bonds in the process of mineralization by Fourier transformed infrared spectroscopy (FTIR; NEXUS 670, Nicolet, WI, USA) over a wavenumber range of 4000–400 cm^−1^. Analyzing the phase compositions of the coatings through X-ray diffractometer (XRD, D/Max-2400, Rigakuco. Ltd. Tokyo, Japan, λ = 0.154181 nm) with Cu Kα radiation at room temperature with wide angles of 2θ from 20° to 80°, the test voltage is 40 kV and the step size is 0.02°.

### 2.6. Corrosion Characterization

#### 2.6.1. Electrochemical Test

Electrochemical measurements were carried out in a standard three-electrode cell in simulated body fluid (SBF, NaCl 7.994 g/L; NaHCO_3_ 0.352 g/L; KCl 0.228 g/L; K_2_HPO_4_ 0.228 g/L; MgCl_2_ 0.306 g/L; CaCl_2_ 0.278 g/L; Na_2_SO_4_ 0.07 g/L) solution at 37 °C using an electrochemistry workstation (Autolab PGSTAT302N, Metrohm, The Netherlands). The samples were served as the working electrode, while Ag/AgCl electrode (saturated with KCl) and a platinum piece were adopted as the reference and counter electrodes, respectively. The exposed area of the working electrode to the electrolyte was 0.5 cm^2^. Before the test, the sample was immersed into SBF for 30 min to obtain a stable open circuit voltage. Potentiodynamic polarization scans were performed at a scanning rate of 1 mV/s. The E_corr_ and i_corr_ were fitted using the Tafel extrapolation method. The electrochemical measurement of each group was repeated three times to confirm the repetition rate of the result.

#### 2.6.2. Hydrogen Evolution Test

The samples were placed into SBF solution of 37 °C, and the funnel connected to the acid burette was inverted to cover the sample completely. Under the condition that the surface was completely exposed, the water level in the burette was measured intermittently for 168 h to gather the total amount of hydrogen evolution and calculate the hydrogen release production per unit area and the corrosion rate. In order to ensure repeatability, three samples were carried out under each condition.

### 2.7. Biocompatibility Evaluation

#### 2.7.1. Materials Preparation

Mouse osteoblast-like cells (MC3T3-E1) (Procell Life Science & Technology Co., Ltd.; Wuhan, China) were used for in vitro biocompatibility measurements, and they were cultured in DMEM medium with 10% fetal bovine serum, 1% streptomycin and penicillin at 37 °C. In this research, the sample extracts were used to study the biocompatibility. Each sample was immersed in complete culture medium for 72 h with a ratio of 3 mL/cm^2^ in accordance with ISO10993−12 [42] to obtain the extracts. The extracts were preserved at 4 °C prior to the experiments.

#### 2.7.2. Cell Viability

Cells were cultured in 96-well plates (3000 cells per well) for 6 h. Then, the complete culture medium was replaced by extracts with equal volume, and cells were cultured for 1,3, and 5 days. The proliferation rates were measured by Cell Counting Kit-8 (CCK-8, Yeasen Biotech Co., Ltd. Shanghai, China).

#### 2.7.3. Live/Dead Assay

The MC3T3-E1 cells were cultured in a 6-well plate and 6000 cells were added to each well. After 6 h, the cell culture medium of each well was replaced by sample extract and cultured at 37 ℃ in a 5% CO_2_ incubator for 1 day. In addition, then the cells were stained by live/dead assay (Yeasen Biotech Co., Ltd. Shanghai, China).

## 3. Results and Discussion

### 3.1. Analysis of CMC-DA

**Figure 1:**
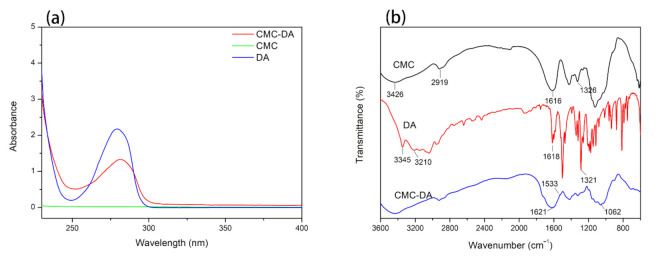
UV–VIS (**a**) and FTIR (**b**) spectra of CMC, DA, and CMC–DA hydrogels.

The UV–VIS spectrum of the samples is displayed in Figure 1a. Due to the presence of catechol groups in its benzene rings, the DA has obvious absorption peak at 280 mm. However, the molecular structure of CMC does not contain the same group, so there was no obvious absorption peak in the range of 225–400 nm [34]. When DA reacts with cellulose, the catechol group is grafted into the molecular chain. In the CMC-DA sample, a characteristic absorption peak similar to that of DA appears around 280 nm. Therefore, the results of the UV–VIS spectroscopy confirm that we successfully prepared a CMC-DA composite hydrogel.

Figure 1b shows the infrared spectrum results of CMC, DA, and CMC-DA. In the CMC spectrum, the peaks at 3426 and 2919 cm^−1^ were caused by O-H bonds and C–H bonds, respectively [36]. Asymmetric and symmetric C=O stretching vibration peaks can be observed at 1616 and 1326^−1^ cm. In the DA sample, the characteristic peak observed near 1618 cm^−1^ is the stretching vibration peak of the C=C bond in the benzene ring, while the 1321 cm^−1^ is the stretching vibration peak of the C–O bond in the catechol structure [43]. In the infrared spectrum of CMC-DA, a new peak appears near the wavenumber of 1621 cm^−1^ due to the superposition of the C=C aromatic peak and the C=O stretching vibration peak. The peak at 1533 cm^−1^ belongs to the N–H bending vibration peak in the newly formed amide bond, and the C–N stretching vibration peak also appears at 1062 cm^−1^. The above results confirm that the amino group in DA reacted with the carboxyl group in carboxymethyl cellulose to form an amide bond, which further verifies the successful synthesis of carboxymethyl cellulose-DA.

### 3.2. Characterization of AZ31, AZ31/CMC-DA, AZ31/HA, and AZ31/CMC-DA/HA Surfaces

**Figure 2:**
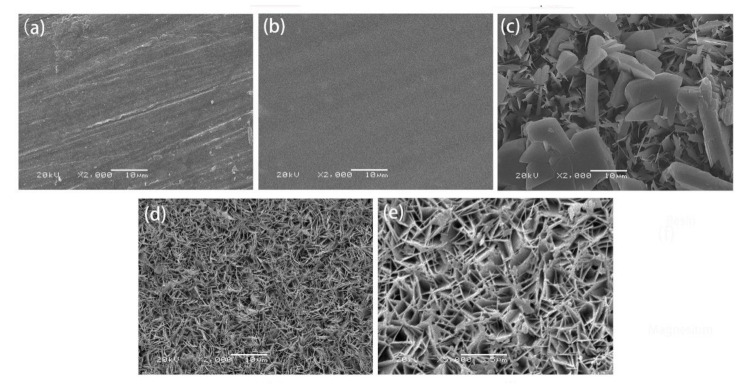
SEM images showing the surface morphologies of (**a**) AZ31, (**b**) AZ31/CMC-DA, (**c**) AZ31/HA, (**d**) AZ31/CMC-DA/HA, and (**e**) AZ31/CMC-DA/HA at a higher magnification.

Surface views of the samples are illustrated in Figure 2a–e. Figure 2a shows that scratches were present on the surface of AZ31 group, which were mainly due to the sandpaper grinding. The number of scratches and cracks on the AZ31/CMC-DA samples was significantly reduced due to the smooth and flat surface of the CMC-DA hydrogel coating. The above results preliminarily confirmed the synthesis of composite hydrogel on the surface of AZ31 alloy. Figure 2c shows the result of mineralization of the AZ31 without surface treatment in a CaP solution. The coating on the surface was disordered and irregular, and large particles had deposited on the surface. It may have been due to the lack of nucleation centers on the surfaces of magnesium alloys and the direct deposition of Ca and P. Figure 2d,e shows a completely different crystal structure from that in Figure 2c. The HA surface induced by CMC-DA was a three-dimensional leaf-like structure, which was similar to the results of Gao et al. [44]. It was composed of porous and uniform flake crystals. The porous structure promotes the biological response of osteocytes, such as inducing the formation of bone apatite, thus stimulating the proliferation of osteoblasts [45]. The uniform leaf-like structure indicates that the addition of CMC-DA hydrogel is beneficial to promote the deposition of HA crystals on the surface of magnesium alloy and the growth in one direction.

**Figure 3:**
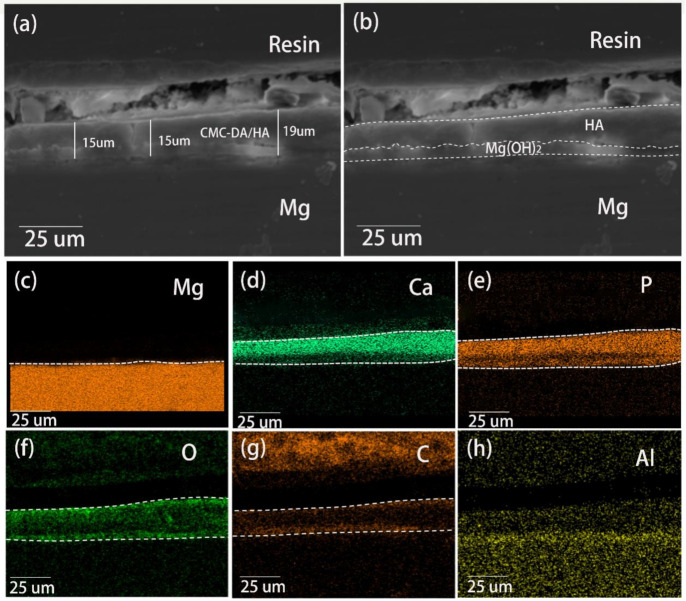
SEM cross-sectional images of (**a**), (**b**) AZ31/CMC-DA/HA and corresponding element distribution of (**c**) Mg, (**d**) Ca, (**e**) P, (**f**) O, (**g**) C, and (**h**) Al.

The cross-sectional morphology of the AZ31/CMC-DA/HA sample is displayed in Figure 3a. The coating’s thickness was measured to be 15.8 ± 0.4 μm. Due to the existence of the induction layer, the adhesion of the CaP coating was improved, and there was no obvious boundary between the magnesium alloy and the coating. Moreover, there was a tentacle-like structure at the bottom of the coating that extended into the surface of the substrate. In addition, the coating showed an obvious double-layer structure as displayed in Figure 3b, which includes internal Mg(OH)_2_ layer and external HA layer [46]. The corresponding element distribution was shown in Figure 3c–h. There are O, Mg, Ca, and P elements in the coating. The presence of Mg and O elements and alkaline environment illustrate that the underlying membrane may be Mg(OH)_2_ coating. The presence of Ca and P elements further illustrates the successful preparation of Ca–P crystals on the surface of magnesium alloys. The surface and cross-sectional analysis results show that not only a regular HA coating is formed on the surface of the magnesium alloy but also the coating has good adhesion to the substrate and a relatively uniform distribution of Ca and P.

**Figure 4:**
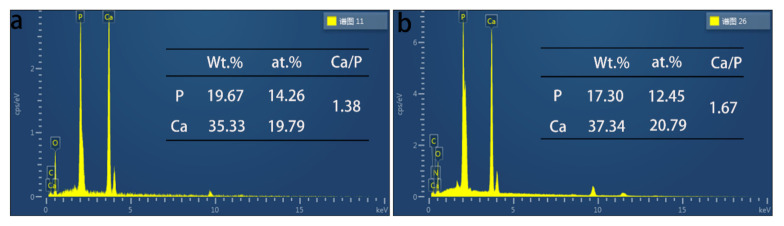
EDS analysis of the elemental compositions on the AZ31/HA (**a**) and AZ31/CMC-DA/HA (**b**) surfaces.

The EDS results are shown in Figure 4. In the AZ31/CMC-DA/HA and AZ31/HA experimental groups, both coatings contain Ca, P, C, and O elements. The existence of oxygen element is mainly caused by the oxidation on the surface of AZ31 alloy [41]. Compared with the AZ31/HA coating, we found the existence of N element in the CMC-DA/HA layer, which mainly comes from the amide bond in the composite layer. According to the specific element content, the Ca/P ratio of the coating directly formed on the surface of magnesium alloy is 1.38, while the Ca/P ratio of CMC-DA-induced HA coating is 1.67, which is more comparable to the Ca/P ratio of pure HA. The results show that the pure HA coating deposited directly lacks calcium ions, which is due to the formation of a small amount of Mg^2+^ ions instead of Ca^2+^ ions in the HA lattice [47]. However, the Ca/P ratio of CMC-DA/HA coating is higher, indicating that more Ca^2+^ ions enter the HA lattice, which plays a positive role in the formation of HA [48]. There is a strong interaction between the carboxyl and hydroxyl groups of CMC-DA and Ca^2+^ ion. Ca^2+^ ion can be adsorbed and rapidly enriched on the hydroxyl-rich surface, which promotes the formation of calcium-rich apatite.

**Figure 5:**
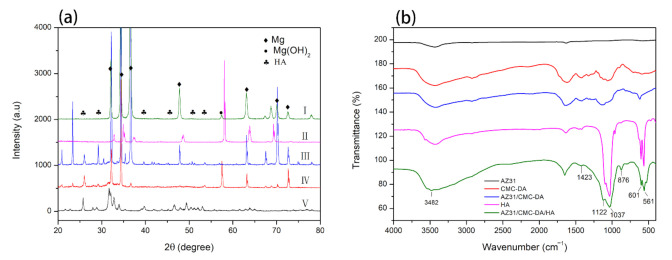
XRD (**a**) spectra of AZ31 I, AZ31/CMC-DA II, AZ31/CMC-DA/HA III, AZ31/HA IV, and pure HA V specimens and FTIR (**b**).

The XRD patterns of pure HA and other groups are shown in Figure 5a. Except for the pure HA samples, all the other samples show obvious α-Mg characteristic peaks, which may be attributed to the porous properties of the coatings [49]. Of course, in the AZ31/CMC-DA group, the characteristic peak of α-Mg was significantly reduced due to the coverage of the composite hydrogel. In addition, the peaks of Mg(OH)_2_ and HA were observed, indicating that the main components of the coating were Mg(OH)_2_ and HA. For AZ31/CMC-DA/HA coating, the characteristic peaks of HA appear at 2 θ = 26°, 39.3°, and 54°, corresponding to (002), (310), and (004) reflection of HA, respectively, confirming the existence of HA phase (according to JCPDS no. 09–0432.) [50]. In addition, the diffraction peak corresponding to HA is narrow, sharp, and symmetrical, indicating that the HA coating has good crystallization [51]. This makes it have a strong adhesion and decrease the degradation of the coating, which will prolong the service life of the implant [52].

Figure 5b explained the FTIR spectrum of the AZ31, CMC-DA, AZ31/CMC-DA, pure HA, and AZ31/CMC-DA/HA coatings. The spectrum shows that the CMC-DA hydrogel was successfully combined with the magnesium alloy. For the AZ31/CMC-DA/HA sample, a sharp peak at 1037 cm^–1^ and a shoulder peak centered at 1122 cm^–1^ was present in the spectra, which were results of the symmetric and asymmetric stretching modes of PO_4_^3–^, respectively [41]. Otherwise, a doublet with one peak at 601 cm^−1^ and another at 561 cm^−1^ was due to the bending vibrations of PO_4_^3–^. Furthermore, CO_3_^2−^ vibration peaks were found at 876 cm^−1^ [53], and the peaks at 1423 and 3482 cm^−1^ were ascribed to the −OH groups in the HA. These peaks confirmed the formation of typical HA and a small content of carbonate-substituted HA [54].

### 3.3. Corrosion Characterization

**Figure 6:**
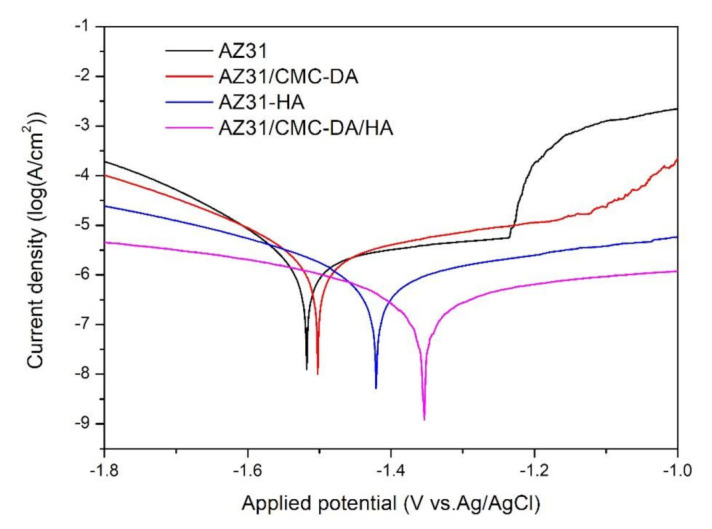
Potentiodynamic polarization (PDP) curves of AZ31, AZ31/CMC-DA, AZ31/HA, and AZ31/CMC-DA/HA specimens in simulated body fluid (SBF).

Figure 6 shows the potentiodynamic polarization curves of all experimental groups, and the corresponding corrosion potential (E_corr_) and corrosion current density (i_corr_) are displayed in Table 1. Because magnesium is easily corroded, the AZ31 sample had a high corrosion current density (i_corr_ = (1.69 ± 0.050) × 10^−6^ A/cm^2^) and low corrosion potential (E_corr_ = −1.53 ± 0.011 V). All the coated samples showed lower i_corr_ and higher E_corr_ numerical values in contrast to those of the bare AZ31 samples. In particular, the AZ31/CMC-DA/HA coating exhibited the lowest i_corr_ value ((1.25 ± 0.003) × 10^−7^ A/cm^2^) and highest E_corr_ (−1.35 ± 0.012 V) of all the coated samples. Compared with uncoated magnesium alloys, the E_corr_ values for the AZ31/CMC-DA/HA positively shifted by approximately 180 mV, and the i_corr_ values decreased 13.5 folds. The main reason was that the composite coating and HA on the surface prevented corrosive ions from penetrating the magnesium alloy surface, reducing the concentration of corrosive ions. According to the above analysis, the biomimetic mineralization of HA induced by cellulose and DA obviously improve the corrosion resistances of magnesium materials.

**Figure 7:**
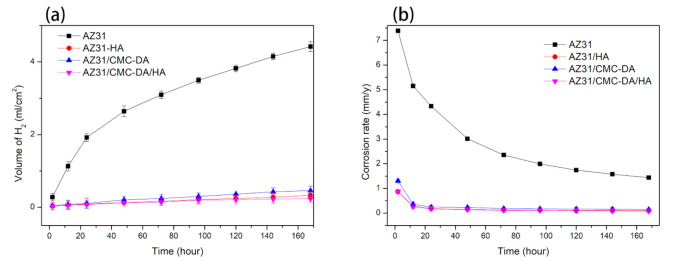
Hydrogen evolution volumes (**a**) and corrosion rate (**b**) of AZ31, AZ31/CMC-DA, AZ31/HA, and AZ31/CMC-DA /HA specimens immersed in SBF for 7 day.

Figure 7 shows the results of hydrogen evolution of all the samples immersed in SBF solution for 7 days. The total amount of hydrogen released by naked AZ31 reached 55 mL within 7 days, while the hydrogen release capacity of AZ31/HA, AZ31/CMC-DA, and AZ31/CMC-DA/HA groups was 4.15, 5.75, and 2.95 mL, respectively. The corrosion rate of AZ31, AZ31/CMC-DA, AZ31/HA, and AZ31/CMC-DA/HA in 7 days are 1.438 ± 0.038, 0.149 ± 0.043, 0.107 ± 0.052, and 0.076 ± 0.051 mm/y, respectively. Naked magnesium AZ31 corroded rapidly when it was first immersed in SBF, releasing a large amount of H_2_. With the increase in soaking time, the corrosion rate decreased gradually because of the synthesis of corrosion products, which plays a protective role on the alloy. In other samples, the protective films greatly increased the corrosion resistance of magnesium materials. In particular, the total hydrogen release amount and release rate of AZ31/CMC-DA/HA samples were the lowest of all samples. In general, potentiodynamic polarization curve and hydrogen evolution spectrum test show that the corrosion resistance of AZ31 coating has been effectively improved.

### 3.4. Cytocompatibility Tests

**Figure 8:**
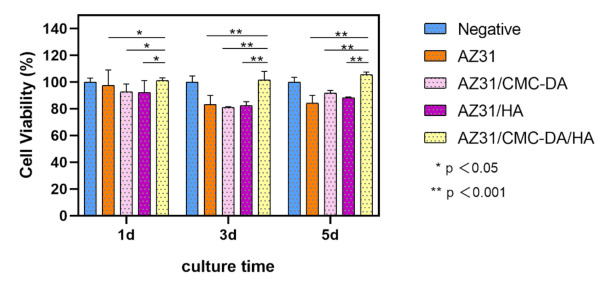
Cell viability of MC3T3-E1 cultured in different extracts prepared with negative, AZ31, AZ31/CMC-DA, AZ31/HA, and AZ31/CMC-DA/HA for 1, 3, and 5 days.

**Figure 9:**
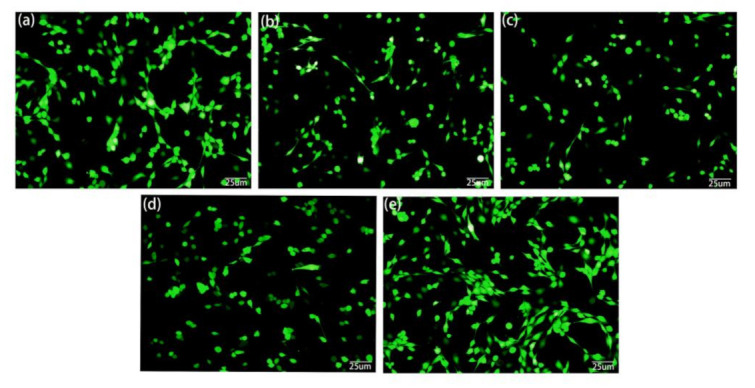
Fluorescent images of MC3T3-E1 after culturing for 24 h in extracts of the (**a**) negative, (**b**) AZ31, (**c**) AZ31/CMC-DA, (**d**) AZ31/HA, and (**e**) AZ31/CMC-DA/HA.

In-vitro cytotoxicity tests are one of the important evaluation methods of the biological properties of biomaterials. The result of cell viability test is shown in Figure 8. On the first day, the survival rate of all MC3T3-E1 cells cultured with extract was more than 90%, which proved that all samples had no obvious cytotoxicity. On the third day, with the increase in culture time, the cell proliferation rate of AZ31, AZ31/CMC-DA, and AZ31/HA groups was lower than that before, which may be due to the exfoliation of the protective film, which led to the corrosion of magnesium alloys and the increase in pH in the culture medium, while in the AZ31/CMC-DA/HA group, the materials showed higher osteogenic activity because of the proliferation-promoting effect of HA on cells. This result is consistent with that of Lin [51]. On the fifth day, the proliferation rate of AZ31/CMC-DA/HA group was significantly higher than that of the other layer groups. At the same time, the living/dead staining results of MC3T3-E1 cells cultured in extract for 24 h are shown in Figure 9a–e. The cells of all samples are generally in a healthy fusiform shape and are widely distributed. The number of cells in AZ31/CMC-DA/HA group was significantly higher than that in other groups and slightly higher than that in negative group. Therefore, the AZ31/CMC-DA/HA group showed good cytocompatibility and enhanced the compatibility with osteoblasts.

## 4. Conclusions

In this study, a novel (CMC-DA/HA) composite coating was prepared by biomimetic mineralization method. As a polymer coating, the carboxyl group and hydroxyl group on the surface of CMC-DA adsorbed calcium and phosphorus and deposited on the surface of magnesium alloy under the electrostatic interaction. The novel coating not only has a dense three-dimensional leaf structure, but also is hydroxyapatite with good crystallinity. Compared with the coating deposited directly on magnesium alloy, it has better properties. The coating can provide good corrosion resistance for magnesium alloy, and HA coating can promote cell proliferation. This study has a good prospect in the field of medical implantation of modified magnesium alloys such as implant anchorage and cardiovascular stents.

## Figures and Tables

**Table 1:** The corrosion potential (E_corr_) and corrosion current density (i_corr_) of the different AZ31 Mg alloy.

Samples	E_corr_ (V vs. Ag/AgCl)	i_corr_ (A/cm^2^)
AZ31	−1.53 ± 0.011	(1.69 ± 0.050) × 10^−5^
AZ31/CMC-DA	−1.51 ± 0.007	(1.52 ± 0.028) × 10^−5^
AZ31/HA	−1.42 ± 0.003	(3.54 ± 0.231) × 10^−6^
AZ31/CMC-DA/HA	−1.35 ± 0.012	(1.25 ± 0.003) × 10^−6^

## Data Availability

The data presented in this study are available on request from the corresponding author.
